# Supplementary material for: All-Trans Retinoic Acid Exhibits Antiviral Effect against SARS-CoV-2 by Inhibiting 3CLpro Activity
Source: Viruses. 2021 Aug 23;13(8):1669. doi: 10.3390/v13081669 (PMC8402917; doi:10.3390/v13081669)
Supplement: Supplementary file 1 [file viruses-13-01669-s001.zip › viruses-1355758-supplementary.pdf]

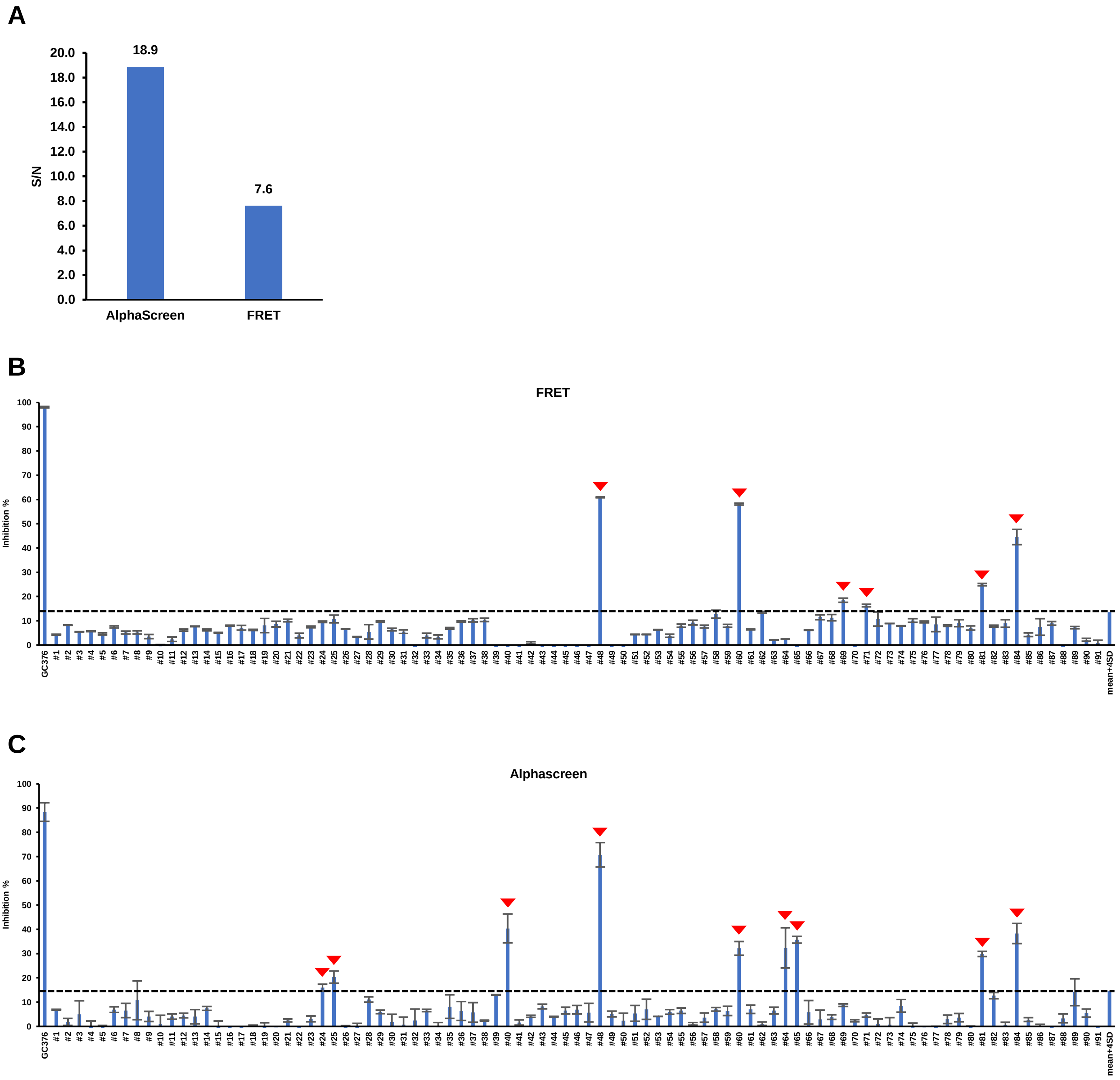

Figure S1. The comparison of FRET and AlphaScreen.

(A) The comparison of sensitivity between FRET and AlphaScreen by S/N ratio.

(B, C) Comparison of FRET and AlphaScreen of compounds used for screening in this experiment by inhibition rate of SARS-CoV-2 3CLpro. The result o fFRET (B) and AlphaScreen (C). Compounds with inhibition rates above the mean +4 SD were marked on these graphs.

Figure S1

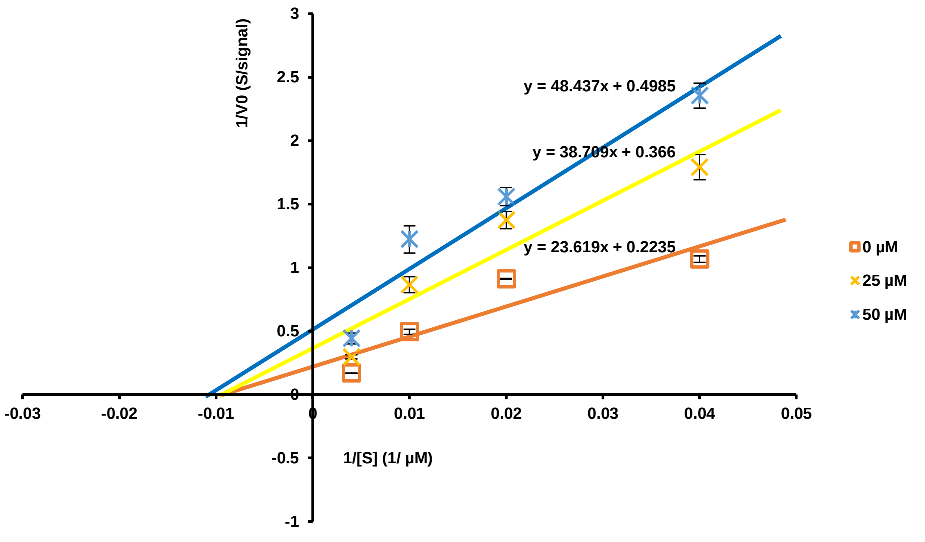

Figure S2. Graphical determination of the type pf inhibition.

Lineweaver-Burk plot of the inhibitory effect of ATRA (0, 25, 50, μM) on SARS-CoV-2 3CLpro at 100 nM.

Figure S2

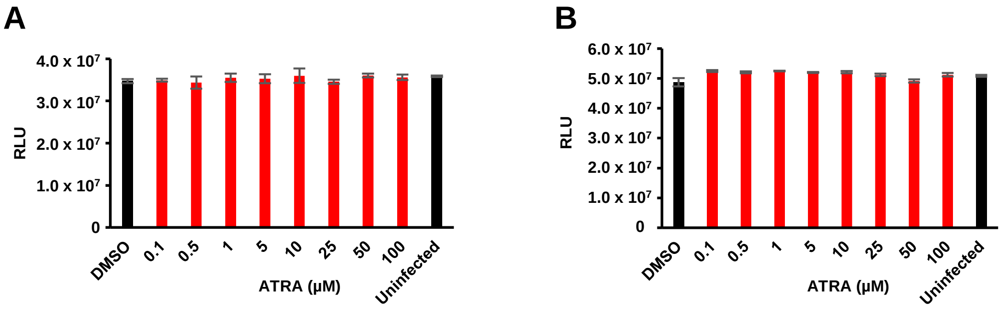

Figure S3. Cytotoxicity of ATRA

(A) Cell viability assay for cytotoxicity at various concentrations of ATRA treated VeroE6/TMPRSS2 cells for 48 hours.

(B) Cell viability assay for cytotoxicity at various concentrations of ATRA treated Calu-3 cells for 72 hours.

Figure S3

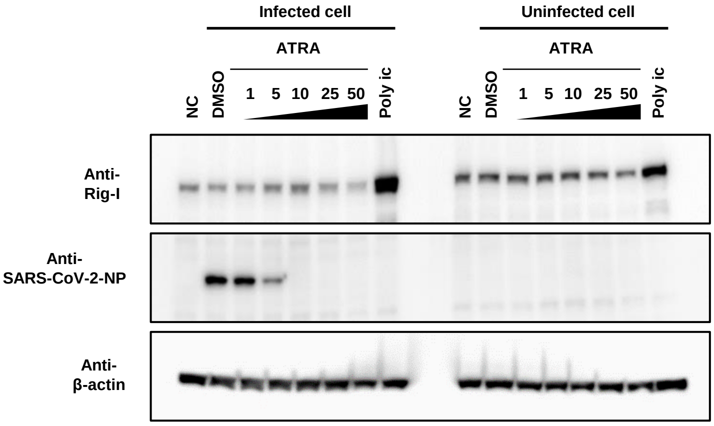

Figure S4. Protein expression in ATRA treated Calu-3 cells during SARS-CoV-2 infection.

Immunoblot analysis of RIG-I, SARS-CoV-2 NP, and β-actin in Calu-3 cell treated with ATRA and Poly I:C.

Figure S4

3CLpro

```
A      SGFRKMAFPSGKVEGCMVQVTCGTTTLNGLWLDDVVYCPRHVICTSEDMLNPNYEDLLIRKSNHNFLVQAGNVQLRVIGHSMQNCVLKLKVDTANPKTPKYKFVRIQPGQTFSVLACYNG 120
Alpha  SGFRKMAFPSGKVEGCMVQVTCGTTTLNGLWLDDVVYCPRHVICTSEDMLNPNYEDLLIRKSNHNFLVQAGNVQLRVIGHSMQNCVLKLKVDTANPKTPKYKFVRIQPGQTFSVLACYNG 120
Beta   SGFRKMAFPSGKVEGCMVQVTCGTTTLNGLWLDDVVYCPRHVICTSEDMLNPNYEDLLIRKSNHNFLVQAGNVQLRVIGHSMQNCVLKLRVDTANPKTPKYKFVRIQPGQTFSVLACYNG 120
Gamma  SGFRKMAFPSGKVEGCMVQVTCGTTTLNGLWLDDVVYCPRHVICTSEDMLNPNYEDLLIRKSNHNFLVQAGNVQLRVIGHSMQNCVLKLKVDTANPKTPKYKFVRIQPGQTFSVLACYNG 120
Delta  SGFRKMAFPSGKVEGCMVQVTCGTTTLNGLWLDDVVYCPRHVICTSEDMLNPNYEDLLIRKSNHNFLVQAGNVQLRVIGHSMQNCVLKLKVDTANPKTPKYKFVRIQPGQTFSVLACYNG 120
       *****************************************************************************************:******************************

A      SPSGVYQCAMRPNFTIKGSFLNGSCGSVGFNIDYDCVSFCYMHHMELPTGVHAGTDLEGNFYGPFVDRQTAQAAGTDTTITVNVLAWLYAAVINGDRWFLNRFTTTLNDFNLVAMKYNYE 240
Alpha  SPSGVYQCAMRPNFTIKGSFLNGSCGSVGFNIDYDCVSFCYMHHMELPTGVHAGTDLEGNFYGPFVDRQTAQAAGTDTTITVNVLAWLYAAVINGDRWFLNRFTTTLNDFNLVAMKYNYE 240
Beta   SPSGVYQCAMRPNFTIKGSFLNGSCGSVGFNIDYDCVSFCYMHHMELPTGVHAGTDLEGNFYGPFVDRQTAQAAGTDTTITVNVLAWLYAAVINGDRWFLNRFTTTLNDFNLVAMKYNYE 240
Gamma  SPSGVYQCAMRPNFTIKGSFLNGSCGSVGFNIDYDCVSFCYMHHMELPTGVHAGTDLEGNFYGPFVDRQTAQAAGTDTTITVNVLAWLYAAVINGDRWFLNRFTTTLNDFNLVAMKYNYE 240
Delta  SPSGVYQCAMRPNFTIKGSFLNGSCGSVGFNIDYDCVSFCYMHHMELPTGVHAGTDLEGNFYGPFVDRQTAQAAGTDTTITVNVLAWLYAAVINGDRWFLNRFTTTLNDFNLVAMKYNYE 240
       ************************************************************************************************************************

A      PLTQDHVDILGPLSAQTGIAVLDMCASLKELLQNGMNGRTILGSALLEDEFTPFDVVRQCSGVTFQ 306
Alpha  PLTQDHVDILGPLSAQTGIAVLDMCASLKELLQNGMNGRTILGSALLEDEFTPFDVVRQCSGVTFQ 306
Beta   PLTQDHVDILGPLSAQTGIAVLDMCASLKELLQNGMNGRTILGSALLEDEFTPFDVVRQCSGVTFQ 306
Gamma  PLTQDHVDILGPLSAQTGIAVLDMCASLKELLQNGMNGRTILGSALLEDEFTPFDVVRQCSGVTFQ 306
Delta  PLTQDHVDILGPLSAQTGIAVLDMCASLKELLQNGMNGRTILGSALLEDEFTPFDVVRQCSGVTFQ 306
       ******************************************************************
```

The catalytic residues **The substrate binding region**

Figure S5. Amino acid sequence alignment of 3CLpro.

Amino acid sequence alignment of 3CLpro from SARS-CoV-2 first isolate strain (lineage A) and VOC; alpha, beta, gamma and delta.

Figure S5

Table S1

No.	Compound name
#1	Delphinidin 3-glucoside chloride
#2	Cyanidin 3-glucoside chloride
#3	Petunidin 3-glucoside chloride
#4	Peonidin 3-glucoside chloride
#5	Delphinidin 3-rutinoside chloride
#6	Cyanidin 3-rutinoside chloride
#7	Delphinidin chloride
#8	Cyanidin chloride
#9	Cyanidin 3-sophoroside chloride
#10	Cyanidin 3-(2G-glucosylrutinoside) chloride
#11	Delphinidin 3-galactoside chloride
#12	Cyanidin 3-galactoside chloride
#13	Petunidin 3-galactoside chloride
#14	Peonidin 3-galactoside chloride
#15	Malvidin 3-galactoside chloride
#16	Pelargonidin 3-glucoside chloride
#17	Delphinidin 3－sambubioside chloride
#18	Delphinidin 3,5-diglucoside chloride
#19	Cyanidin 3-sambubioside-5-glucoside chloride
#20	Cyanidin 3-sambubioside chloride
#21	Delphinidin 3-arabinoside chloride
#22	Procyanidin B-1
#23	Hyperoside
#24	Pteropodine (Uncalin C)
#25	Isopteropodine (Uncalin E)
#26	6-Hydroxygenistein 6,7-diglucoside
#27	Tectorigenin 7-o-xylosylglucoside
#28	Licochalcone A
#29	Daidzin
#30	Daidzein
#31	Glycitin
#32	Glycitein
#33	Genistin
#34	Genistein
#35	Xanthohumol
#36	Isoquercitrin
#37	Luteolin
#38	(+)-Catechin
#39	(-)-Epicatechin
#40	(-)-Epigallocatechin
#41	(-)-Epicatechin gallate
#42	(-)-Epigallocatechin gallate
#43	3,5,7,3',4'-Pentamethoxyflavone
#44	5,7,4'-Trimethoxyflavone
#45	5,7-Dimethoxyflavone
#46	3,5,7-Trimethoxyflavone
#47	3,5,7,4'-tetramethoxyflavone
#48	All-trans-retinoic acid
#49	Liquiritin
#50	Liquiritigenin
#51	Glabridin
#52	Hesperidin
#53	Neohesperidin
#54	Calycosin 7-O-Glucoside
#55	Quercetin 3-O-[2''-O-(6'''-O-p-Coumaroyl)-b-D-Glucopyranosyl]-a-L-Rhamnopyranoside
#56	Quercetin 3-O-[2''-O-b-D-Glucopyranosyl]-a-L-Rhamnopyranoside
#57	(-)-Gallocatechin 3-O-(3''-O-methyl)gallate
#58	Isoliquiritin
#59	Ganoderic acid A
#60	Corosolic acid
#61	Soyasapogenol B
#62	18β-Glycyrrhetinic acid
#63	Bilobalide
#64	Ginkgolide A
#65	Ginkgolide B
#66	Ginkgolide C
#67	Glycyrrhizinic acid (Glycyrrhizin)
#68	Soyasaponin Ⅰ
#69	Soyasaponin Ⅴ
#70	Deacylgymnemic acid
#71	Ginsenoside Rg1
#72	Tenuifolin
#73	Withaferin A
#74	Tectoridin
#75	Tectorigenin
#76	Rhynchophylline
#77	Verbascoside (acteoside)
#78	Arctiin
#79	Echinacoside
#80	Isoacteoside
#81	6-Gingerol
#82	8-Gingerol
#83	10-Gingerol
#84	6-Shogaol
#85	Cryptochlorogenic acid
#86	Neochlorogenic acid
#87	Isochlorogenic acid A
#88	Isochlorogenic acid B
#89	Sennoside A
#90	Sennoside B
#91	Mangiferin
